# The reliability of the plethysmographic perfusion index for detecting fluid responsiveness in critically ill patients

**DOI:** 10.1016/j.jointm.2025.07.004

**Published:** 2025-09-27

**Authors:** Younes Aissaoui, Ayoub Bouchama, Chaimae Abouelkemhe, Abdellah Enourhbi, Bassam Bencharfa, Mehdi Didi, Hatim Aguena, Ayoub Belhadj

**Affiliations:** Cadi Ayyad University Faculty of Medicine and Pharmacy of Marrakech, Marrakesh, Morocco

**Keywords:** Fluids, Acute circulatory failure, Hemodynamic monitoring, Pulse oximetry, Gray zone analysis

## Abstract

**Background:**

The assessment of fluid responsiveness (FR) is critical during the initial management of acute circulatory failure (ACF). However, in resource-limited settings or emergency situations, advanced hemodynamic monitoring is often unavailable. The plethysmographic perfusion index (PPI), a non-invasive parameter derived from pulse oximetry, has been proposed as a surrogate marker for changes in cardiac output. The objective of this study was to evaluate the ability of the PPI to detect FR in critically ill patients with ACF during early resuscitation.

**Methods:**

This was a prospective observational study conducted over seven months (February to September 2024) in a 10-bed intensive care unit. Adult patients with ACF requiring a 500-mL fluid bolus were enrolled. FR was assessed by transthoracic echocardiography, defined as *a* ≥ 15% increase in left ventricular outflow tract velocity-time integral (VTI). VTI and PPI values were obtained before and immediately after the completion of fluid administration. The diagnostic performance of changes in PPI (ΔPPI) was assessed using receiver operating characteristic (ROC) curve analysis and a gray zone approach.

**Results:**

Fifty patients (29 males and 21 females) were included, with a median age of 65 years (interquartile range [IQR]: 57–77). Sepsis-related ACF accounted for 60% of cases. FR was observed in 33 patients (66%). The area under the ROC curve (AUC) for ΔPPI was 0.778 (95% CI: 0.638 to 0.883, *P* = 0.004). A ΔPPI threshold of 33% yielded 70% sensitivity and 82% specificity. The gray zone ranged from 0% to 88%, encompassing 30% of the cohort.

**Conclusion:**

The PPI demonstrated moderate accuracy in detecting FR during early resuscitation of ACF. As a simple, non-invasive, and widely accessible tool, PPI holds promise for informing fluid management decisions in emergency and critical care settings. Further technological refinements may enhance its diagnostic performance and broaden its clinical applicability.

**Trial registration** Clinical Trials.gov Identifier: NCT06313671.

## Introduction

Fluid resuscitation is a cornerstone in the management of acute circulatory failure (ACF). However, only approximately half of patients respond to fluid administration with a significant increase in cardiac output.^[1]^ Consequently, it is essential to accurately assess fluid responsiveness (FR) in patients with ACF, as both fluid overload and insufficient resuscitation can lead to adverse outcomes, including tissue hypoperfusion and organ dysfunction.^[2]^ Several techniques have been developed for predicting FR, ranging from invasive methods such as pulse contour analysis to non-invasive approaches such as echocardiographic indices.^[3]^ However, these tools often require specialized equipment and expertise, which limits their applicability during the early phases of resuscitation, such as in emergency settings or resource-limited environments.

The plethysmographic perfusion index (PPI) is derived from the ratio of pulsatile to non-pulsatile light absorption measured via photoplethysmography.^[4^^,^^5]^ It has gained growing interest in perioperative and critical care settings as a tool for hemodynamic management, assessment of peripheral perfusion, and prediction of outcome.[4], [5], [6], [7], [8], [9], [10] Because stroke volume (SV) is a key determinant of the PPI, changes in this index (ΔPPI) may reflect changes in SV, making it a potential surrogate for assessing FR.^[4]^ Indeed, an increase in the PPI following a fluid bolus is expected to reflect an increase in SV, thereby aiding in FR determination.

However, changes in PPI observed over longer periods (e.g., from one day to the next) may also reflect alterations in the non-pulsatile component, which can be influenced by factors such as venous congestion or tissue edema.^[4]^ Although a few studies have explored the diagnostic value of the PPI in this context, evidence remains limited, particularly in critically ill patients.[11], [12], [13], [14], [15], [16] The aim of this study was to evaluate the ability of the PPI to detect FR in ACF. We hypothesized that changes in PPI during a fluid bolus would reflect changes in SV.

## Methods

### Study design and ethics

This was a prospective, single-center, observational study conducted over seven months (February to September 2024) in a 10-bed intensive care unit (ICU) of a university-affiliated hospital. The study received approval from the institutional review board, and informed consent was obtained from the legal representatives of the patients. The study was registered on ClinicalTrials.gov (NCT06313671) and adhered to Standards for Reporting of Diagnostic Accuracy Studies (STARD) guidelines.^[17]^

### Participants

Patients aged 18 years or older with ACF were eligible for inclusion if fluid resuscitation was planned to improve hemodynamic status. ACF was defined as the presence of tissue hypoperfusion, including prolonged capillary refill time greater than 3 s, mottling, oliguria, elevated lactate (>2 mmol/L), or metabolic acidosis.^[18]^ All patients were enrolled during the optimization phase of fluid resuscitation for ACF.^[19]^

Patients were excluded if they presented with poor echogenicity, acute cor pulmonale, or absent plethysmographic signals. Poor echogenicity was defined as the inability to obtain a valid measurement of the left ventricular outflow tract velocity-time integral (VTI).

### Procedures

All patients underwent standard hemodynamic monitoring, including invasive arterial pressure measurement when indicated, as well as echocardiographic assessment. Fluid resuscitation consisted of a 500-mL bolus of 0.9% saline or Ringer’s lactate, administered over 15 to 20 min. VTI and PPI measurements were recorded before and immediately after fluid administration.

### PPI measurement

The PPI was measured using the plethysmographic module of the Mindray Beneview T6 monitor. The sensor was placed on the second, third, or fourth finger, and the site with the highest baseline PPI value was selected. Measurements were avoided during vasopressor dose adjustments to prevent fluctuations. PPI values were recorded as raw, real-time readings without temporal averaging.

### PPI precision assessment

To assess the reproducibility of PPI measurements, their precision and least significant change (LSC) were evaluated in a subgroup of 15 patients during a period without hemodynamic fluctuations, defined as <10% variation in systolic arterial blood pressure or catecholamine dosage. Four successive PPI values were recorded for each patient. The coefficient of variation (CV) was calculated as the standard deviation divided by the mean of the four measurements. The coefficient of error (CE) was then computed as the CV divided by the square root of the number of replicates ($\sqrt{n}$). Precision was defined as 2 × CE, and the LSC, representing the smallest change not attributable to measurement variability, was calculated as LSC = CE × 1.96 × $\sqrt{2}$.^[11^^,^^20]^

### Echocardiographic measurements

Transthoracic echocardiographic examinations were performed by two board-certified and experienced critical care echocardiographers (YA and AB) following international guidelines.^[21]^ A LOGIQ e ultrasound system (GE Healthcare, California, USA) equipped with a GE-3S RS phased-array probe was used for all evaluations.

VTI was measured at end-expiration, averaged over three cardiac cycles, and obtained in the apical five-chamber view using pulsed-wave Doppler, as recommended.^[20]^ The standard parameters of left and right ventricular systolic and diastolic function were also recorded.^[22]^

### Definition of fluid responsiveness

FR was defined as *a* ≥15% increase in VTI after fluid infusion, consistent with current recommendations.^[1^^,^^23]^

### Data collection

ΔVTI and ΔPPI after fluid administration were calculated as follows:

ΔVTI=(Post−FluidVTI−BaselineVTI)/BaselineVTI

ΔPPI=(Post−FluidPPI−BaselinePPI)/BaselinePPI

Data collected included patient characteristics, hemodynamic and echocardiographic variables, therapeutic interventions, and triggers prompting fluid administration.^[23]^ Skin tone was assessed for each patient using the Fitzpatrick classification, which categorizes skin phototypes from I (very pale) to VI (very dark).^[24]^ For subgroup analysis, patients were grouped into light (types I–III) and dark (types ≥IV) skin tone categories.

### Statistical analysis

Categorical variables were reported as counts (percentages), and continuous variables as the mean ± standard deviation or the median with interquartile ranges (IQRs), depending on data distribution. Normality was assessed using the Shapiro–Wilk test. For comparisons between groups, the unpaired Student’s *t*-test or the Mann–Whitney *U* test was applied for continuous variables, while for categorical variables, the chi-squared test was used.

Correlations between ΔPPI and ΔVTI were assessed using Spearman’s correlation coefficient. A four-quadrant plot analysis was performed to assess the directional agreement between ΔPPI and ΔVTI following fluid administration. An exclusion zone was applied using the LSC for each parameter to account for measurement variability and avoid misclassification of clinically insignificant changes. The LSC for ΔPPI was calculated based on repeated measurements and is reported in the Results section. The LSC for ΔVTI was set at 6%, according to a previous study by the same authors.^[25]^ who also reported an excellent interobserver agreement for VTI measurements in this setting.^[26]^

The ability of ΔPPI to detect FR was assessed using receiver operating characteristic (ROC) curve analysis with 95% confidence intervals (CIs). The optimal ΔPPI cut-off for detecting FR was determined using Youden’s index, and the corresponding sensitivity, specificity, positive predictive value (PPV), and negative predictive value (NPV) were reported.

A gray zone analysis was conducted to identify inconclusive ΔPPI values, defined as thresholds where sensitivity and specificity were both below 90%.^[27]^ The ability of ΔPPI to detect FR was compared across subgroups, including mechanically ventilated *vs.* spontaneously breathing patients, patients receiving *vs.* not receiving vasopressors, those with darker (Fitzpatrick >III) *vs.* lighter (Fitzpatrick ≤III) skin tones, and those with axillary temperatures ≥36 °C *vs.* <36 °C. Differences in the areas under the curves (AUCs) between these subgroups were evaluated using the DeLong test for correlated ROC curves.

Based on an expected FR incidence of 50%^[28]^ and an AUC of 0.75, a minimum of 50 patients was required to ensure acceptable diagnostic accuracy with a precision of 0.1.

All statistical analyses were conducted using MedCalc version 14.8 (MedCalc, Ostend, Belgium), SPSS version 26.0 (IBM, Armonk, NY, USA), and Jamovi version 2.3.28 (The Jamovi Project, 2022). RStudio (Posit PBC, Boston, MA, USA) was used for generating the quadrant plot. A *P*-value of <0.05 was considered statistically significant.

## Results

### Patient characteristics

**Figure 1:**
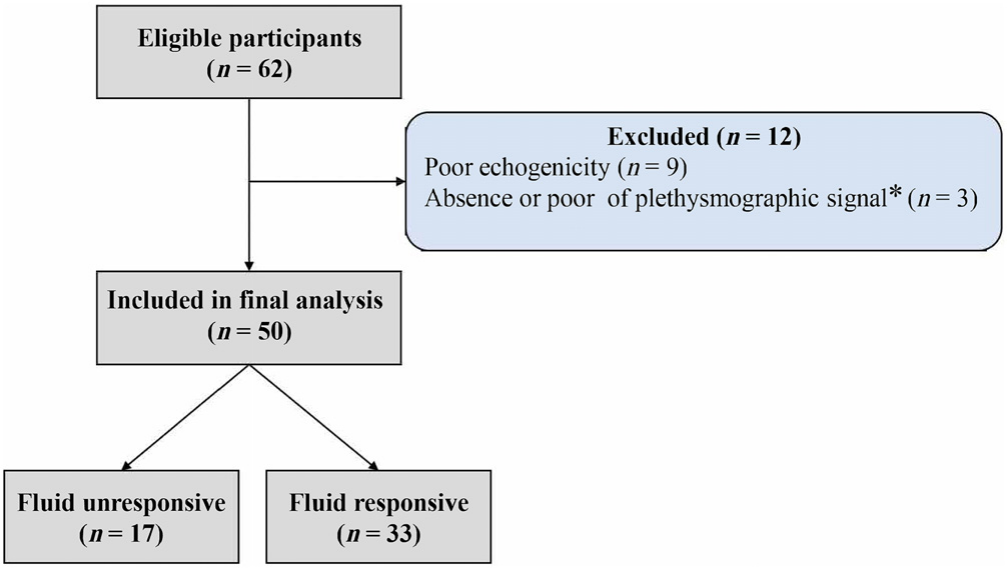
Flow chart of the study. *One patient had henna on thumbs.

During the study, a total of 62 patients met the inclusion criteria (Figure 1). Nine patients were excluded due to poor echogenicity, and three due to the absence of a plethysmographic signal. For one of these three patients, the absence of a signal was attributed to the presence of henna dye on the thumbs, which likely interfered with sensor detection. Consequently, 50 patients were included in the final analysis. The median delay between ACF diagnosis and patient inclusion was 6 h (IQR: 3–13).

The median age of the patients was 65 years (IQR: 57–77), and 42.0% were female (Table 1). The median Acute Physiology and Chronic Health Evaluation II (APACHE II) score was 21 (IQR: 15–26), and the median Charlson Comorbidity Index was 5 (IQR: 3–6). Diabetes and arterial hypertension were present in 40% and 34% of patients, respectively. The most common reasons for ICU admission were septic shock (32%), sepsis (26%), and postoperative care (20%). The highest number of ACF cases was septic in origin (60%), followed by hemorrhagic and hypovolemic non-hemorrhagic causes, accounting, respectively, for 22% and 14% of cases. The most frequent triggers for fluid administration were arterial hypotension (82%), hyperlactatemia (74%), skin hypoperfusion (50%), and oliguria (46%). Twenty-two patients (44%) were under invasive mechanical ventilation.

**Table 1:** Overview of patient characteristics.

Characteristics	Data (*n* = 50)
Age (years)	65 (57–77)
Female sex	21 (42)
APACHE II score	21 (15–26)
Charlson comorbidity index	5 (3–6)
Comorbidities	
Diabetes	20 (40)
Arterial hypertension	17 (34)
Chronic kidney disease	8 (16)
Chronic obstructive pulmonary disease	7 (14)
Reason for ICU admission	
Septic shock	16 (32)
Sepsis	13 (26)
Postoperative admission	10 (20)
Neurologic causes	7 (14)
Acute respiratory failure (without shock)	2 (4)
Severe trauma	2 (4)
Acute circulatory failure etiology	
Septic	30 (60)
Hemorrhagic	11 (22)
Hypovolemic non-hemorrhagic	7 (14)
Cardiogenic	2 (4)
Triggers for fluid administration	
Arterial hypotension	41 (82)
Skin hypoperfusion (mottling or CRT >3 s)	25 (50)
Altered mental status	16 (32)
Oliguria	23 (46)
Hyperlactatemia	37 (74)
Skin tone (I/II/III)*	3/5/19
Skin tone (IV/V/VI)*	20/1/2

Data are presented as median (interquartile range) or *n* (%)*.*

⁎ Fitzpatrick classification of skin tone: I (very pale), II (very fair), III (fair), IV (olive/light brown), V (dark brown), VI (very dark or black). APACHE II: Acute physiology and chronic health evaluation II; CRT: Capillary refill time; ICU: Intensive care unit; PLR: Passive leg raising.

### Assessment of PPI precision

The CV of the PPI was 9.0%, with a CE of 4.5%, corresponding to a precision of 9.0%. The LSC was 12.5%, representing the minimum variation required to reflect a true physiological change beyond measurement variability.

### Comparison between fluid-responsive and fluid-non-responsive patients

Overall, 33 (66%) patients were fluid responders. Hemodynamic parameters, including heart rate, arterial pressure, and norepinephrine use, were comparable between responders and non-responders (Table 2). The volume of fluids administered also did not significantly differ between the two groups (median = 1000 [IQR: 500–2000] mL *vs.* median = 1500 [IQR: 1000–2075] mL, *P* = 0.217). Blood lactate levels were significantly higher in responders than in non-responders (median = 3.4 [IQR: 2.8–5.4] mmol/L *vs.* median = 2.0 [IQR: 1.3–2.1] mmol/L, *P* = 0.001). Mechanical ventilation parameters were similar between the two groups (*P* = 0.772).

**Table 2:** Baseline hemodynamic, echocardiographic, and management variables in fluid responders and non-responders.

Parameters	Fluid responders (*n* = 33)	Non-responders (*n* = 17)	*P* value
Heart rate (beats/min)	108±25	105±29	0.687
Systolic arterial pressure (mmHg)	113±14	109±21	0.385
Diastolic arterial pressure (mmHg)	62±9	61±12	0.752
Mean arterial pressure (mmHg)	79±7	75±15	0.274
Pulse pressure (mmHg)	51±17	49±19	0.530
Volume of fluids administered (mL)	1000 (500–2000)	1500 (1000–2075)	0.217
Norepinephrine infusion	21 (63)	8 (47)	0.242
Norepinephrine dosage (µg/ (kg·min))	0.32 (0.19–1.1)	0.35 (0.04–0.45)	0.573
Blood lactate level (mmol/L)	3.4 (2.8–5.4)	2.0 (1.3–2.1)	0.001
Mechanical ventilation	15 (45)	7 (41)	0.772
Tidal volume (mL/kg of IBW)	6.4 (6.1–7.3)	6.8 (6.0–7.4)	0.729
Plateau pressure (mmHg)	20 (17–25)	19 (18–24)	0.829
Driving pressure (mmHg)	13 (9–16)	14 (11–16)	0.458
PEEP (cmH_2_0)	5 (5–9)	5 (5–8)	0.362
PaO_2_/FiO_2_ (mmHg)	180 (120–260)	254 (175–293)	0.556
VTI before fluid (cm)	11.3 (8.6–13.3)	11.9 (10.7–16.6)	0.187
VTI after fluids (cm)	14.1 (11.3–17)	12.7 (10.3–17.4)	0.510
Fluid-induced changes in VTI (%)	27 (19–39)	4 (−5–10)	<0.0001
PPI before fluids	0.83 (0.36–1.41)	0.65 (0.40–1.57)	0.905
PPI after fluids	1.19 (0.43–1.83)	0.59 (0.41–1.14)	0.193
Fluid-induced changes in PPI (%)	60 (17–108)	1 (−28–31)	0.001
E wave (cm/s)	65 (49–74)	75 (59–98)	0.015
A wave (cm/s)	72 (51–83)	75 (47–100)	0.732
E/A ratio before fluids	0.8 (0.7–0.9)	1.5 (0.8–1.4)	0.043
E/e′ ratio	5.8 (4.7–6.9)	7.7 (5.8–11.5)	0.035
E wave (cm/s) after fluids	65 (49–74)	75 (59–98)	0.015
A wave (cm/s) after fluids	72 (51–83)	75 (47–100)	0.732
E/A ratio after fluids	0.8 (0.7–0.9)	1.5 (0.8–1.4)	0.043
E/e′ ratio after fluids	5.8 (4.7–6.9)	7.7 (5.8–11.5)	0.035
MAPSE (mm)	14 (11–16)	13 (10–16)	0.327
LVEF (%)	59 (50–64)	55 (42–67)	0.703
TAPSE (mm)	20 (19–24)	18 (12–23)	0.116

Continuous data are reported as mean ± standard deviation or median (interquartile range) and categorical data as *n* (%).

A: Atrial peak velocity of transmitral flow with pulsed Doppler; e′: Early diastolic peak velocity of the mitral annulus, measured by tissue Doppler imaging. The value used is the mean of the lateral and septal annular velocities; E: Early peak velocity of transmitral flow with pulsed Doppler; FiO_2_: Inspired fraction of oxygen; IBW: Ideal body weight; LVEF: Left ventricular ejection fraction; MAPSE: Mitral annular plane systolic excursion; PaO_2_: Partial arterial pressure of oxygen; PEEP: Positive end-expiratory pressure; PPI: plethysmographic perfusion index; TAPSE: Tricuspid annular plane systolic excursion; VTI: Velocity-time integral of the left ventricular outflow tract.

Before fluid administration, VTI values were comparable between the two groups (*P* = 0.187). However, fluid-induced changes in VTI were significantly greater in responders (median = 27% [IQR: 19%–39%]) than in non-responders (median = 4% [IQR: −5%–10%]) (*P* <0.0001). Fluid-induced changes in the PPI were also higher among responders (median = 60% [IQR: 17%–108%]) than among non-responders (median = 1% [IQR: −28%–31%]) (*P* = 0.001). Baseline and post-fluid PPI values were not significantly different between the groups (*P* >0.05).

Compared to non-responders, fluid responders had significantly lower baseline E-wave velocities, E/A ratios, and mean E/e′ ratios, both before and after fluid administration (all *P*<0.05) (Table 2). This suggested that baseline left ventricular filling pressure was lower in the latter group. No significant differences in the systolic indices of the left and right ventricles were observed between the two groups (Table 2).

### The ability to change in the PPI after fluid administration to detect FR

**Figure 2:**
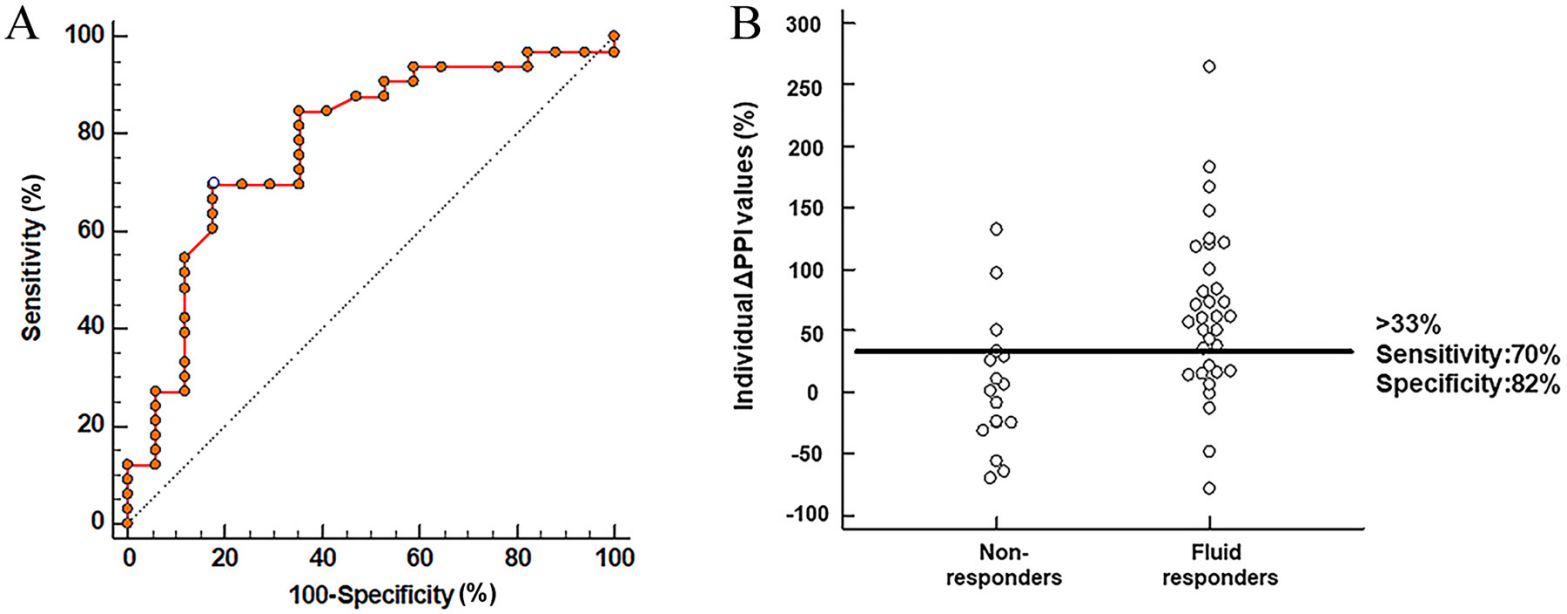
Receiver operating characteristic curve (A) evaluating the ability of fluid-induced changes in the ΔPPI to detect fluid responsiveness: B: Individual ΔPPI values for responders (*n* = 33) and non-responders (*n* = 17). ΔPPI: Plethysmographic perfusion index.

The area under the ROC curve (AUC) for ΔPPI was 0.778 (95% CI: 0.638 to 0.883, *P* = 0.004) (Figure 2A). A ΔPPI threshold of 33% detected FR with a sensitivity of 70% (95% CI: 51% to 84%) and a specificity of 82% (95% CI: 57% to 96%) (Figure 2B). The corresponding PPV was 89% (95% CI: 7%–99%), whereas the NPV was 58% (95% CI: 37% to 78%) (Table 3).

**Table 3:** Diagnostic performance and threshold analysis of ∆PPI for the detection of fluid responsiveness in patients with acute circulatory failure.

Items	∆PPI (%)	Sensitivity (%)	Specificity (%)	PPV (%)	NPV (%)
Optimal threshold value	33	70 (51 – 84)	82 (57 – 96)	89 (7 – 99)	58 (37 – 78)
Upper limit of gray zone	88	27 (13 – 47)	90	83 (50 – 98)	39 (23 – 55)
Lower limit of gray zone	0	90	47 (23 – 72)	77 (61 – 89)	67 (35 – 90)

Values are expressed with 95% confidence intervals, except for the specificity of the upper limit and the sensitivity of the lower limit of the gray zone.

NPV: Negative predictive value; PPI: Plethysmographic perfusion index; PPV: Positive predictive value.

The correlation between ΔVTI and ΔPPI was not statistically significant, with a correlation coefficient of 0.142 (95% CI: −0.070 to 0.337, *P* = 0.161).

**Figure 3:**
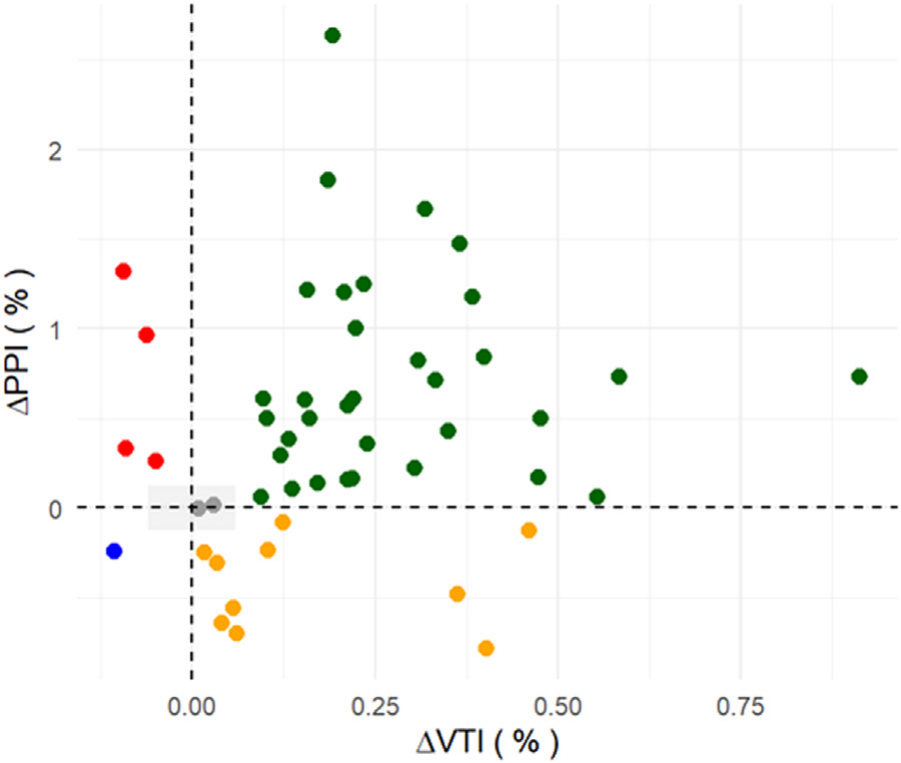
Quadrant plot showing the directional agreement between changes in the ΔPPI and changes in ΔVTI after fluid administration. The gray box represents the exclusion zone based on the LSC (6% for ΔVTI and 12.5% for ΔPPI). Concordant increases in both ΔVTI and ΔPPI were observed in 70% of patients. Green dots indicate true positives (concordant responders), yellow dots indicate false negatives, red dots indicate false positives, the blue dot indicates a true negative, and gray dots represent cases within the exclusion zone. LSC: Least significant change; ΔPPI: Plethysmographic perfusion index; ΔVTI: Velocity-time integral.

The four-quadrant plot (Figure 3), incorporating exclusion zones based on the LSCs for ΔVTI (6.0%) and ΔPPI (12.5%), demonstrated a moderate-to-strong directional concordance for these parameters. Among evaluable patients, 70% exhibited concordant changes, with simultaneous increases or decreases in both ΔVTI and ΔPPI, suggesting that there was a reasonable agreement between changes in the PPI and SV variations following fluid administration.

### Subgroup comparisons

The performance of ΔPPI in detecting FR varied across clinical subgroups. It was higher in patients receiving norepinephrine (AUC = 0.848, 95% CI: 0.655 to 1.040, *P* <0.0001) than in those not on vasopressors (AUC = 0.750, 95% CI: 0.502 to 0.998, *P*=0.048). Similarly, mechanically ventilated patients exhibited slightly better ΔPPI performance (AUC = 0.833, 95% CI: 0.649 to 1.018, *P* <0.0001) than spontaneously breathing patients (AUC = 0.771, 95% CI: 0.566 to 0.975, *P*=0.009). When exploring the influence of skin tone, ΔPPI showed better FR discriminating ability in patients with darker skin tones (Fitzpatrick >III, AUC = 0.878, 95% CI: 0.626 to 1.000) than in those with lighter tones (Fitzpatrick ≤III, AUC = 0.595, 95% CI: 0.343 to 0.847). Finally, ΔPPI also performed better in patients with an axillary temperature ≥36 °C (AUC = 0.857, 95% CI: 0.674 to 1.040, *P* <0.0001) than in those with temperatures <36 °C (AUC = 0.589, 95% CI: 0.293 to 0.885, *P*=0.555) (*P*=0.132). Although these findings suggested that vasopressor use, mechanical ventilation, skin tone, and temperature may potentially influence ΔPPI performance, none of the subgroup comparisons reached statistical significance.

### Gray zone analysis

**Figure 4:**
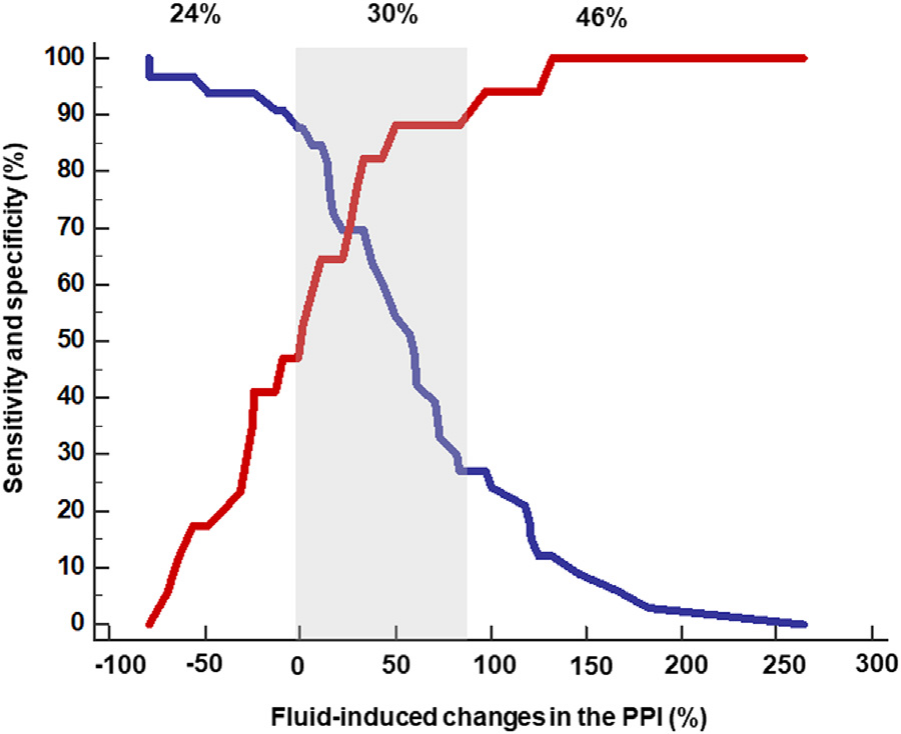
Gray zone analysis of the ability of fluid-induced changes in the plethysmographic perfusion index (ΔPPI) to detect fluid responsiveness. The red curve represents sensitivity, and the blue curve represents specificity. Percentages indicate the proportion of patients within the conclusive and inconclusive zones. ΔPPI: Plethysmographic perfusion index.

A gray zone analysis identified an inconclusive range between 0% and 88%, encompassing 15 patients (30% of the cohort) (Figure 4). At the upper limit (ΔPPI = 88%), specificity was 90%, while sensitivity was 27% (95% CI: 13% to 47%). At the lower limit (ΔPPI = 0%), sensitivity reached 90%, with a specificity of 47% (95% CI: 23% to 72%) (Table 3).

## Discussion

This prospective study demonstrated that the PPI provides a moderate but clinically meaningful ability to detect FR during the early management of ACF. As a simple, non-invasive, and widely accessible parameter, PPI may serve as a useful adjunct for guiding fluid resuscitation when advanced hemodynamic monitoring is unavailable. Ours is among the few prospective studies to evaluate ΔPPI using a fluid bolus combined with echocardiographic reference measurements.

From a physiological perspective, the PPI reflects the ratio between the pulsatile and non-pulsatile components of the photoplethysmographic waveform. The non-pulsatile component is mainly influenced by venous return and peripheral tissue characteristics, while the pulsatile component is related to arterial pulsations. Given that the PPI is determined based on the interaction between vascular tone and SV.^[4^^,^^5]^, rapid changes in this index may reflect corresponding changes in SV.

Several studies have investigated the diagnostic performance of the PPI in identifying FR, with reported accuracies ranging from modest to high depending on patient populations, monitoring techniques, and clinical contexts.[10], [11], [12], [13], [14], [15], [16], [17] Beurton et al.^[11]^ evaluated ΔPPI during a passive leg raising (PLR) maneuver in a mixed ICU population and reported an AUC of 0.89 with a threshold of 9% (Sensitivity 89%, Specificity 88%). In a subsequent study, the same team assessed changes in the PPI during an end-expiratory occlusion test and found a similarly high AUC of 0.95, but with a lower threshold of 2.5%.^[14]^ In another study, Bruscagnin et al. ^[15]^ assessed ΔPPI during a tidal volume challenge and reported excellent diagnostic accuracy, with significantly higher performance recorded when the PPI was measured on the forehead (AUC = 0.98, cut-off = 18%) than when it was measured on the fingertip (AUC = 0.85, cut-off = 12%).

A common feature across these studies was that PPI values were recorded from averaged data (12 to 30 s average) rather than being real-time bedside measurements.^[11^^,^^14^^,^^15]^ Although this method may enhance signal stability and explain their superior diagnostic performance compared to our findings, it also limits direct applicability in routine clinical practice. Moreover, all three studies used calibrated pulse contour analysis as the reference method for FR, which, although precise, is not widely available in all ICUs.^[11^^,^^14^^,^^15]^ Finally, although forehead sensors appear to improve diagnostic accuracy, they may not be readily accessible in resource-limited settings, which further supports the need for pragmatic, bedside-applicable approaches. It is also worth noting that these studies were conducted in heterogeneous patient populations.

Unlike earlier studies, Lian et al. ^[13]^ evaluated ΔPPI in a more homogeneous population of patients with septic shock. They reported an AUROC similar to ours (0.776) with a ΔPPI threshold of 5% for FR detection after 250 mL to 750 mL fluid loading. Hasanin et al. ^[12]^ also focused on septic shock patients and used transthoracic echocardiography to assess FR after a 200-mL fluid challenge, reporting an AUC of 0.82 with a ΔPPI threshold close to ours (33%) (Sensitivity 76%, Specificity 73%). More recently, Casazzo et al. ^[16]^ included patients with sepsis and severe malaria in a low-income setting. Using echocardiography combined with a PLR test, they reported a baseline AUROC of 0.87 with a ΔPPI threshold of 9%. However, the predictive performance of ΔPPI significantly declined over time, falling to 0.73 at 6 h, and continued to fall until 72 h post-admission. This trend suggests that ΔPPI may be a more reliable predictor of FR in the very early phase of resuscitation. The moderate accuracy observed in our study aligns with this finding, especially considering that the median time to PPI measurement in our cohort was approximately 6 h after ACF diagnosis. Importantly, we identified a cut-off value of 33% for detecting FR using ΔPPI, which is substantially higher than the LSC of 12.5% calculated for our cohort. This indicates that the observed changes in the PPI are likely to represent true physiological responses rather than random measurement variation.

In our study, no significant correlation was found between ΔPPI and ΔVTI following fluid administration, contrasting with several other studies that reported modest to strong correlations between changes in the PPI and SV.^[11^^,^^14^^,^^15]^ However, most of these studies used short, reversible preload challenges such as PLR or ventilatory maneuvers, and often relied on averaged PPI data. In contrast, fluid boluses over 15 to 20 min, as used in our study, may introduce additional variability through changes in vasomotor tone and sympathetic activity, both of which are known to independently influence the PPI.^[10]^ These methodological differences, along with variability in reference standards (e.g., echocardiography *vs*. pulse contour analysis), preload challenge type, timing of assessment, patient populations, vasopressor use, and the specific monitoring devices used (e.g., Mindray, Masimo, Dräger), likely explain the discrepancies in diagnostic performance across studies.[10], [11], [12], [13], [14], [15], [16] Despite the lack of a statistically significant correlation, the four-quadrant plot demonstrated 70% directional concordance between ΔPPI and ΔVTI, supporting the ability of ΔPPI to reflect SV changes after fluid loading. While the precision of the PPI (9%) in our study was lower than that reported by Beurton et al.,^[11]^ it remained within an acceptable range for clinical interpretation and reflects real-world measurement variability under typical ICU conditions.

ΔPPI showed superior performance in patients receiving norepinephrine and those under mechanical ventilation, although the difference was not statistically significant. These trends may reflect physiological mechanisms. Vasopressors can improve peripheral perfusion, enhancing the pulsatile signal of the PPI, while mechanical ventilation may stabilize intrathoracic pressure and reduce respiratory variability in cardiac output. However, the response of the PPI to vasopressors is not consistent. For instance, He et al. ^[29]^ reported variable and sometimes contradictory changes in the PPI following norepinephrine-induced increases in blood pressure, highlighting the complex interplay between the macro- and microcirculation. These findings emphasize that PPI is a context-sensitive parameter and should be interpreted in light of vasopressor use, ventilatory status, skin temperature, and skin tone.

To better define the clinical applicability of ΔPPI, we performed a gray zone analysis, revealing an inconclusive range between 0% and 88%, encompassing 30% of patients. In clinical practice, values above 88% suggest FR, while values below 0% indicate likely non-responsiveness. For intermediate values, additional clinical or hemodynamic assessment is warranted. To date, the only other study that applied a gray zone analysis to ΔPPI was conducted by de Courson et al. ^[10]^ in a perioperative setting and using a lung recruitment maneuver. They identified a gray zone encompassing 26% of patients. These findings highlight that, although ΔPPI can aid in guiding fluid management, its performance is limited by a wide range of diagnostic uncertainty.

Our study had several limitations. First, it was a single-center study with a relatively small sample size, which may limit the generalizability of the findings. Although the sample size was calculated to ensure adequate diagnostic precision, larger multicenter studies are needed to validate these results across more diverse ICU populations. Notably, most of the studies cited here for comparison had similarly small cohorts.[11], [12], [13], [14], [15], [16] Second, the inclusion of both medical and surgical ICU patients introduced heterogeneity; however, this reflects real-world practice and enhances external validity. Finally, we used a fluid challenge to assess FR. While clinically relevant, this method is non-reversible, unlike PLR or ventilatory maneuvers. Accordingly, giving fluids to non-responder patients may expose them to unnecessary volume loading.

## Conclusions

The PPI demonstrated moderate but clinically meaningful accuracy in detecting FR during the early management of ACF. As a simple, non-invasive, and widely accessible tool, PPI holds promise for informing FR decisions, particularly in emergency and resource-limited settings. Future technological refinements and integration with other hemodynamic indicators may further enhance its diagnostic performance and clinical utility.

## CRediT authorship contribution statement

**Younes Aissaoui:** Writing – review & editing, Writing – original draft, Visualization, Validation, Supervision, Software, Resources, Project administration, Methodology, Investigation, Formal analysis, Data curation, Conceptualization. **Ayoub Bouchama:** Writing – review & editing, Software, Resources, Investigation, Formal analysis, Data curation. **Chaimae Abouelkemhe:** Writing – review & editing, Investigation, Formal analysis, Data curation, Conceptualization. **Abdellah Enourhbi:** Investigation, Data curation. **Bassam Bencharfa:** Writing – review & editing, Investigation, Data curation. **Mehdi Didi:** Investigation, Data curation. **Hatim Aguena:** Writing – review & editing, Investigation, Data curation. **Ayoub Belhadj:** Writing – review & editing, Writing – original draft, Validation, Supervision, Methodology, Investigation, Formal analysis, Data curation, Conceptualization.
